# Etiology and risk factors associated with a pruritic papular eruption in people living with HIV in India

**DOI:** 10.7448/IAS.16.1.17325

**Published:** 2013-09-03

**Authors:** Terry T Farsani, Sachin Kore, Patrick Nadol, Mandalaparthy Ramam, Sara J Thierman, Kieron Leslie, Chockalingam Chandrasekar, Rajasekaran Sikhamani, Gurusamy Manoharan, Asha Kubba, Toby A Maurer

**Affiliations:** 1 Harvard University School of Medicine, Boston, MA, USA; 2 Government Hospital of Thoracic Medicine (GHTM), Chennai, India; 3 International Training and Education Center for HIV (Itech), Chennai, India; 4 All India Institute for Medical Sciences (AIIMS), New Delhi, India; 5 Department of Dermatology, State University of New York Downstate Medical Center, New York, NY, USA; 6 Department of Dermatology, University of California San Francisco, San Francisco, CA, USA

**Keywords:** papulopruritic eruption, dermatologic, skin, AIDS, HIV, CD4, eosinophilic folliculitis

## Abstract

**Introduction:**

Papulopruritic eruption (PPE) occurs in people living with HIV in India. Understanding the risk factors associated with this disease may help decrease the prevalence of PPE.

**Methods:**

This study was a case-control study performed at the Government Hospital of Thoracic Medicine, a tertiary care hospital in Chennai, India. Cases included HIV-positive, antiretroviral (ARV) therapy-naïve adults experiencing a pruritic skin eruption for longer than one month, with evidence of multiple papular or nodular lesions and biopsy consistent with arthropod bite. Controls included HIV-positive, ARV-naïve patients without active skin rash. Main outcome measures were CD4 cell count, histology, and environmental exposures. We performed statistical analysis using Epi Info version 3.5.1 and SPSS version 11.0 (SPSS Inc., Chicago, IL). Categorical variables such as gender, urban versus rural residence, occupation, treatment history, CD4 count, use of insect repellents, and environmental exposures were evaluated using the χ^2^ test (or the Fisher exact test when an expected value for a category was less than 5). The *t*-test was used to evaluate differences in age and the duration since HIV diagnosis. The Mann-Whitney test was used to compare non-normally distributed values such as CD4 cell count. A *p*-value that was less than 0.05 was considered to be statistically significant.

**Results:**

Forty-one cases and 149 control subjects were included. Subjects with PPE had significantly lower CD4 cell counts compared to controls (225.5 cells/µL vs. 425 cells/µL; *p*=0.0001). Sixty-six percent of cases had a CD4 cell count less than 350 cells/µL. PPE cases were less likely to use mosquito repellent techniques (odds ratio 2.81, CI = 1.45–5.45).

**Discussion:**

PPE may be an altered and exaggerated immune response to arthropod bites in HIV-positive patients. CD4 cell count is significantly lower in patients with PPE, and therefore it may be considered a qualifying clinical finding for ARV initiation in resource-poor settings. Protective measures against mosquito bites appeared to be important in preventing PPE in subjects at risk.

## Introduction

“Papulopruritic eruption” (PPE) of HIV is a major cause of morbidity in AIDS patients in Asia. It is a commonly occurring, intensely pruritic, and stigmatizing skin rash that is reported to occur in 12–46% of patients with HIV infection, varying by geographic location [1–4]. In patients with PPE, average CD4 counts have been reported to range between 46 and 165 cells/µL [3,5–11]. Beginning in 1983, studies from African countries [1,2,12–16], India [8,17], Thailand [18,19], China [20], and Brazil [9] described an extremely pruritic, diffuse skin eruption occurring in HIV-positive patients. The primary lesion for PPE is a firm, discrete, erythematous, urticarial papule or pustule originating on the extremities and occasionally evolving onto the trunk [3]. Most patients scratch the lesions because of the severe pruritus, leading to excoriated papules, marked postinflammatory pigment changes, and, eventually, prurigo-like nodules. The persistent pruritus is usually refractory to topical steroids and oral antihistamines [13]. However, in a group of patients with PPE, pruritus decreased with the use of antiretroviral (ARV) therapy within a 16-week period [6].

A study from Uganda showed that PPE was histologically consistent with arthropod bites. Whether PPE is due to an actual insect bite or a reaction process that histologically mimics an arthropod bite is unclear. This histologic reaction pattern may also be observed in lymphomatous malignant processes, as well as benign pseudolymphomatous disorders, which may occur in response to various factors, including insect bites and medications. Ultimately, if a lymphomatous process is being entertained, proper histological stains should be performed to help rule out malignant processes. Although mechanisms of disease were not directly demonstrated, PPE is thought to be a hypersensitivity reaction to arthropod bites and is associated with immunodeficiency [7,21].

HIV surveillance in India estimates that the national HIV prevalence is 0.36% (0.30–0.50%), with the majority of infections concentrated in high-risk populations such as commercial sex workers, men who have sex with men, and intravenous drug users. The geographic burden of the disease is reported to be heterogeneous, with HIV prevalence highest in the southern and northeastern states, ranging from 0.03% in Himachal Pradesh to 1.67% in Manipur [22]. Common papular eruptions specific to HIV-positive individuals include PPE and eosinophilic folliculitis (EF). Clinically, these disorders are difficult to distinguish from one another, as they both consist of extremely pruritic generalized papules and pustules involving the face, trunk, and extremities. Histopathologic examination may help aid in the differentiation of PPE from EF by examination of serial sections, which demonstrate normal pilosebaceous units with a lymphocytic- and eosinophilic-rich perivascular infiltrate in the former, in comparison to a folliculocentric lymphocytic and eosinophilic infiltrate in the latter [7,21]. In addition, EF histopathologically demonstrates spongiosis of the follicular epithelium, with the infiltrate concentrated near the follicular isthmus and sebaceous duct. Another histopathological comparison may include suppurative folliculitis. In this disorder, neutrophils and macrophages predominate in the infiltrate, and microorganisms are readily found in the inflammatory reaction. Involved follicles in suppurative folliculitis are notably ruptured [21].

Information about the relationship between PPE and HIV/AIDS in India is limited, although a study in south India found that PPE was seen in almost 8% of HIV-positive patients visiting a non-governmental care and treatment center [17]. In a recent study, 22.5% of newly diagnosed HIV-positive patients visiting a governmental tertiary-care hospital in Jagdalpur were found to have PPE [23]. The prevalence of EF in HIV-positive individuals also varies widely; it was observed in 3.1% of HIV-positive patients in Mumbai in one study and in 11.7% of infected individuals in Bangkok [19,24].

We performed a case-control study in a tertiary-care government hospital in south India to better describe HIV-positive patients with PPE and to identify potential risk factors for PPE in HIV-positive patients seeking services there. We describe the histology of PPE lesions, and compare the HIV profiles as well as environmental risk factors in PPE cases versus non-PPE controls.

## Methods

This study consisted of two phases. In the first phase, we recruited HIV-positive patients with PPE and performed a cross-sectional, descriptive study to determine the prevalence and etiology of PPE in HIV-positive patients. In the second phase, we recruited HIV-positive patients without PPE, and we compared them to the previously recruited HIV-positive patients with PPE to assess risk factors that may be associated with this skin disease.

The study was performed at the Government Hospital of Thoracic Medicine (GHTM), a tertiary care hospital in Chennai, India. GHTM is India's largest publicly supported HIV care and treatment facility center. GHTM has been providing HIV/AIDS care since 1990 and ARV therapy since 2004, and it is recognized for its care and treatment of people living with HIV and AIDS. An estimated 300–500 HIV-positive patients visit GHTM each day for HIV-related care, treatment, and counseling.

This study was carried out with approval from institutional review boards at the University of California, San Francisco (UCSF), and GHTM. All subjects were provided information from a trained study investigator about the methodology, information to be collected, potential risks, and purpose of the study in their preferred language (Tamil, English, or Telugu). After counseling and prior to enrollment, each patient provided written consent to be part of the study. Verbal consent was obtained for taking photographs and for clinical use as well as study purposes. All records were entered into a secure, electronic database (Epi Info v. 3.5.1 [Centers for Disease Control and Prevention]) for analysis.

### Part I

Patient screening and enrollment were carried out over a 12-week period from April to June 2008. Known HIV-positive, ARV-naïve patients, 18 years of age and older, who were being evaluated by trained hospital staff at GHTM as part of standard HIV-related services, were queried regarding pruritic rash originating on extremities for a duration longer than one month. If they met these criteria, they were examined by an Indian-trained dermatologist for clinical confirmation of PPE and a 4.0 mm punch biopsy was taken from a skin lesion that both the patient and the dermatologist identified as the newest and most representative lesion of PPE. Specimens were prepared in New Delhi and sent to the All India Institute of Medical Sciences (AIIMS), New Delhi. Histologic slides were read by an Indian-trained dermatopathologist with previous experience in HIV dermatopathology, including PPE. The dermatopathologist was blinded to the on-site clinical diagnoses. Subjects included as cases were those who met clinical criteria for PPE with histologic confirmation. Histologic confirmation of PPE was defined as a moderately dense to dense, superficial and deep, perivascular and interstitial infiltrate of lymphocytes and eosinophils beneath the epidermis, consistent with “insect bite reaction.” Serial sections were examined to exclude a folliculocentric pathologic process, including EF.

Rash severity was evaluated using an objective “rash-severity scale” for PPE that was created for this study. Lesions that were limited to either the upper or lower extremities were described as “mild.” When both the upper and lower extremities were involved, the rash was categorized as “moderate” disease. A “severe” rash included lesions present on the extremities as well as the trunk. Lesions present all over the body, including the face, were defined as “very severe.”

In order to assess for potential risk factors, subjects meeting inclusion criteria were administered an oral and detailed questionnaire in their preferred language (Tamil, Telugu, or English) by trained clinicians. The questionnaire included items assessing the history of the rash, environmental and other types of exposures, and prior history of skin disorders (Table 1).

**Table 1:** Questionnaire administered to all subjects in their preferred language

Questionnaire
Screening question: Are you currently experiencing a skin problem of greater than one month duration?
Environmental exposure(s)
I. Do you have animals? If yes, what type?
II. Do you use: 1. mosquito netting; 2. insect repellents; 3. insect coils?
Other exposure(s)
I. Do you live in an urban or rural area?
II. What is your main occupation?
III. Does this work occur mostly indoors or outdoors?
IV. At what time of day do you work?
Arthropod exposure
I. How many bug bites do you see on your skin on a daily basis?
II. What do you think normally bites you?
Prior skin disease history
I. Have you had any prior skin problems, including eczema, psoriasis, scabies, or a skin reaction to medicines or drugs?

Routinely collected demographic and clinical data from the electronic THIS (TB and HIV Information System) database at GTHM were obtained for each subject with regard to immunologic status, which included WHO status, CD4 count, and date of HIV diagnosis. The THIS electronic patient database was implemented in 2001 at GHTM and collects routine and longitudinal data for patients during each visit to the facility using a unique patient identification number that is generated at the initial patient visit. This secure system stores and maintains patient data and can be accessed to inform patient care as well as to generate program-level reporting to the facility and other stakeholders.

### Part II

To identify potential risk factors related to PPE, we recruited a control group, which included known HIV-positive, ARV-naïve patients older than 18 years of age, who did not have an active skin rash at the time of screening. Patients who reported experiencing skin lesions or pruritis, or who had any evidence of skin lesions on clinical exam, were excluded from this part of the study. Patient screening and enrollment were carried out over a one-week period in October 2008. After the same consent process as described in the “Part I” section was performed by trained hospital staff, the same questionnaire discussed in “Part I” was orally administered in the patient's preferred language (Table 1).

### Statistical analysis

We performed statistical analysis using Epi Info version 3.5.1 and SPSS version 11.0 (SPSS Inc., Chicago, IL). Similar data were collected for both the PPE subjects and the control populations. Unique outcome measures included the histological characteristics of new pruritic lesions in the study population. Categorical variables such as gender, urban versus rural residence, occupation, treatment history, CD4 count, use of insect repellents, and environmental exposures were evaluated using the χ^2^ test (or the Fisher exact test when an expected value for a category was less than 5). The *t*-test was used to evaluate differences in age and the duration since HIV diagnosis. The Mann-Whitney test was used to compare non-normally distributed values such as CD4 cell count. A *p*-value of less than 0.05 was considered to be statistically significant.

## Results

**Figure 1:**
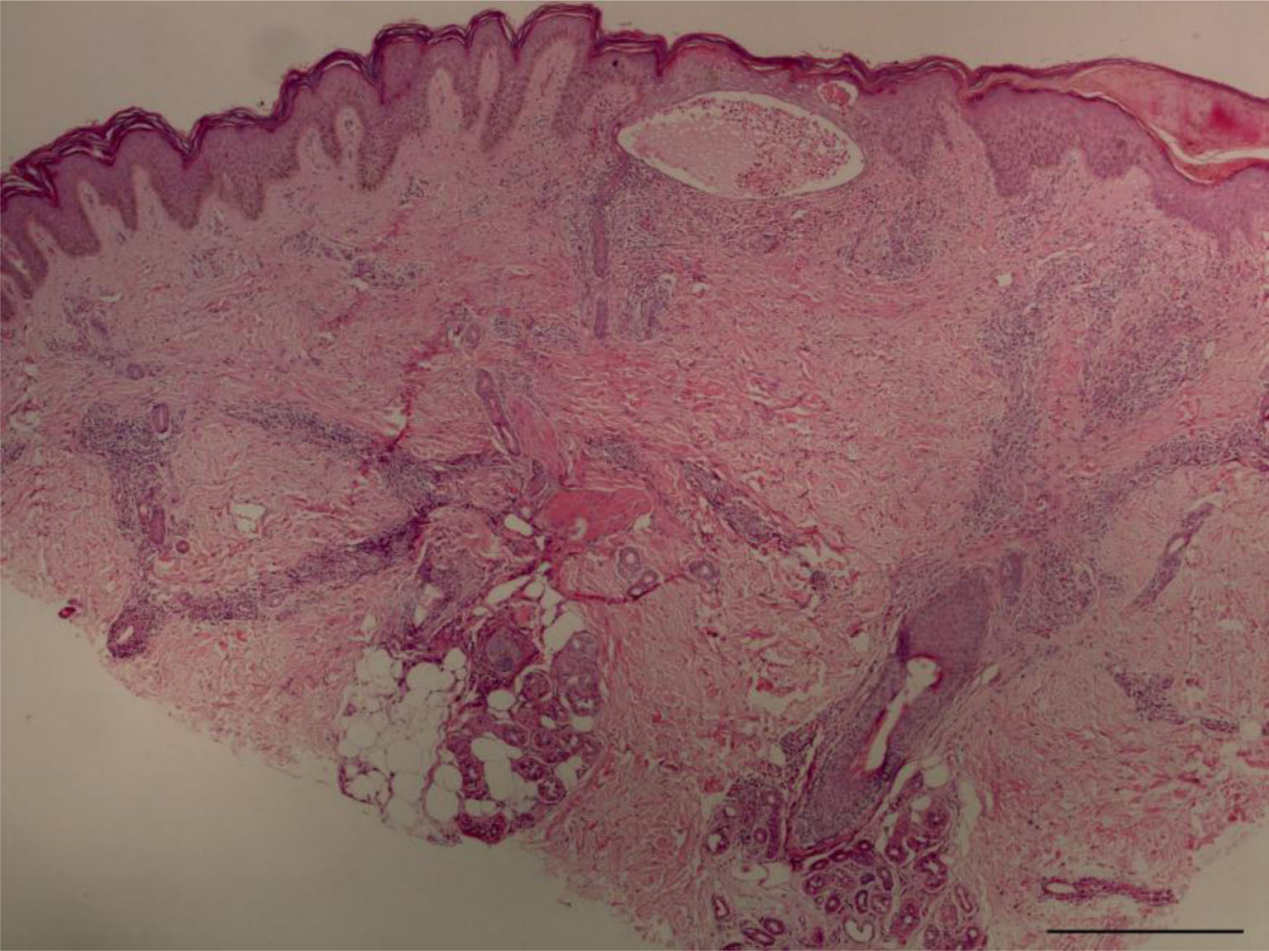
Skin biopsy showing fibrin in the stratum corneum, an intraepidermal vesicle, and a perivascular and interstitial dermal infiltrate (H&E, 40×).

**Figure 2:**
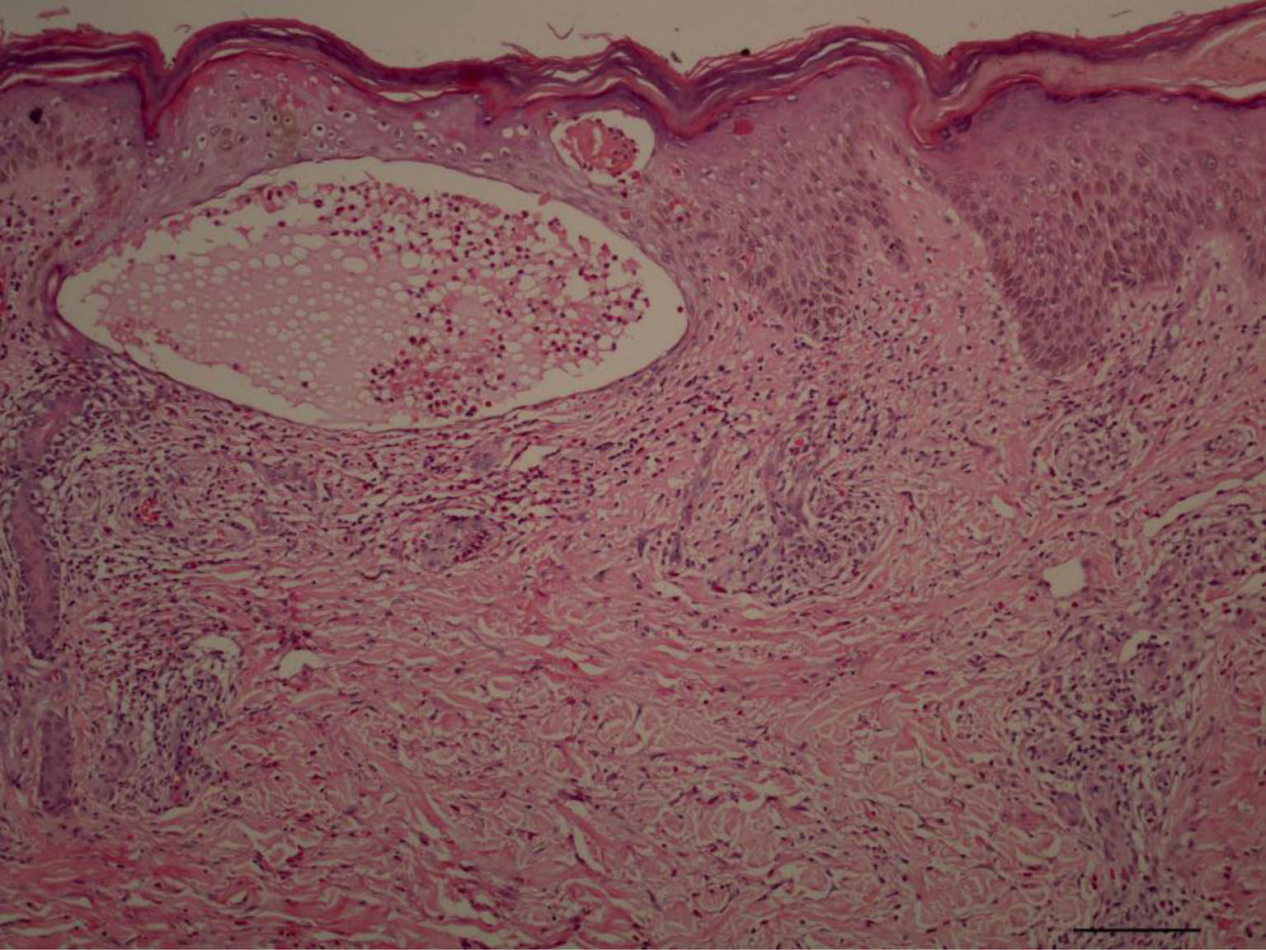
Spongiotic intraepidermal vesicle with an underlying perivascular and interstitial dermal infiltrate (H&E, 100×).

**Figure 3:**
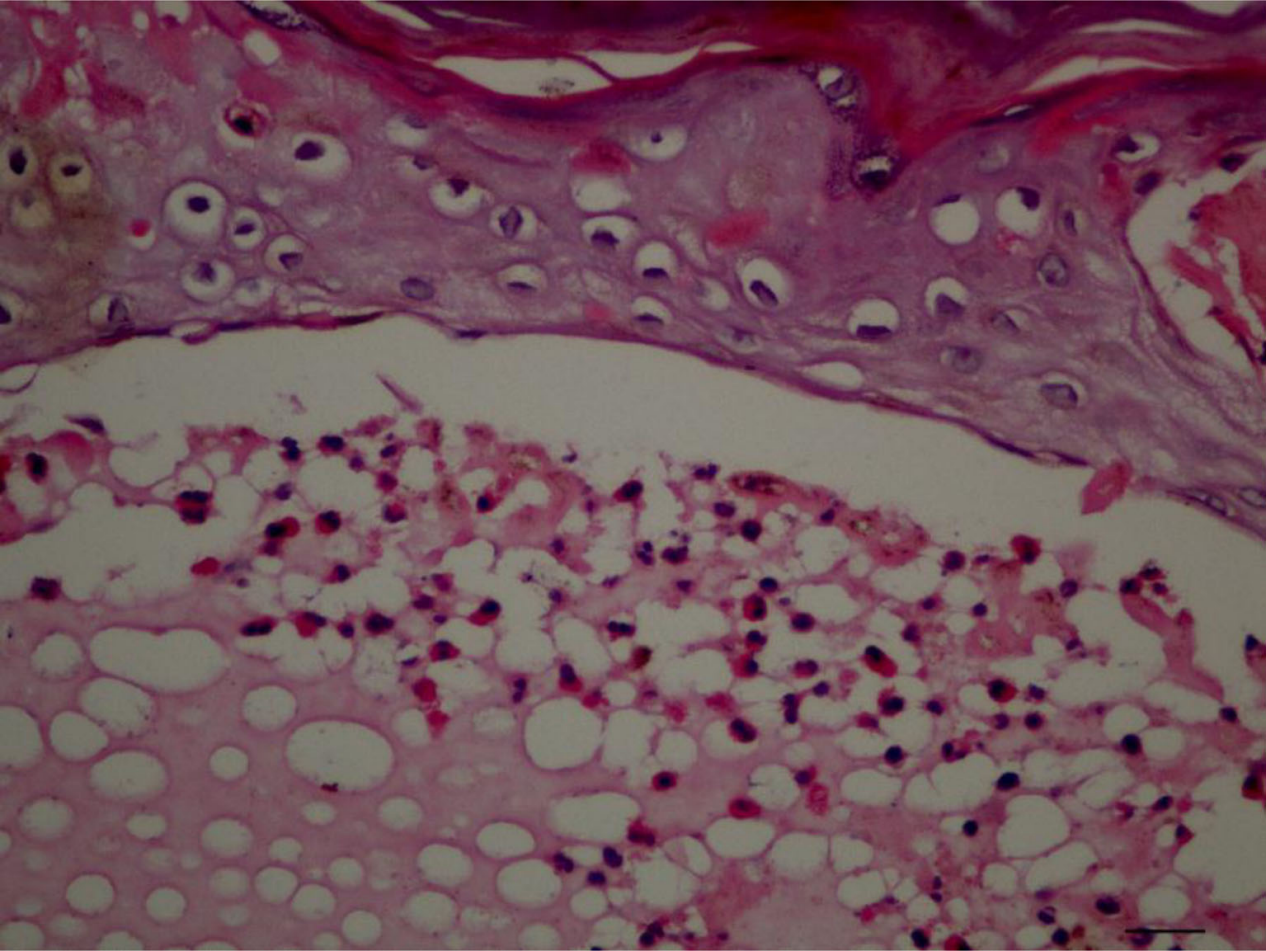
Fibrin and eosinophils are seen within the intraepidermal vesicle (H&E, 400×).

**Figure 4:**
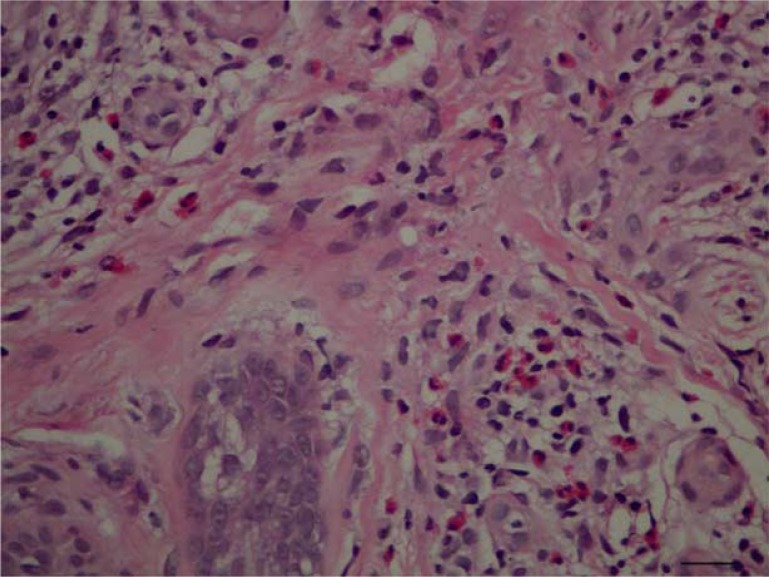
Eosinophils are prominent in the dermal infiltrate (H&E, 400×).

From April to June 2008, a total of 52 subjects who self-reported a pruritic skin eruption for longer than one month duration, with a clinical exam consistent with PPE, were recruited from both the outpatient and inpatient populations at GHTM. Of these 52 patients, 42 (79.2%) were found to have histologically confirmed PPE, and they were included in our final analysis (Figures 1–4).

This represented 2.9% of all patients screened (*n*=1466) during this time period at GHTM. The remaining biopsies were consistent with other histologic diagnoses (Table 2).

**Table 2:** Skin biopsy findings in enrolled subjects (total *n*=52), classified by type of reaction and analyzed by gender (Fisher's exact text = 0.154)

	Sex		
		
Biopsy findings	Female *n* (%)	Male *n* (%)	*n* (%)
Arthropod bite reaction	29 (82.9)	13 (72.2)	42 (79.2)
Psoriasiform	3 (8.6)	0 (0)	3 (5.7)
Granulomatous	1 (2.9)	1 (5.6)	2 (3.8)
Excoriation	0 (0)	1 (5.6)	1 (1.9)
Spongiotic dermatitis	1 (2.9)	0 (0)	1 (1.9)
Non-diagnostic	1 (2.9)	2 (66.7)	3 (5.7)

Using the rash severity scale created for this study, patients with PPE were described as having a mild (*n*=1 [1.9%]), moderate (*n*=16 [30.8%]), severe (*n*=29 [55.8%]), or very severe (*n*=6 [11.5%]) rash. Increasing rash severity was associated with lower CD4 cell counts, but this trend was not statistically significant (*p*=0.7). The majority (*n*=34, 66%) of confirmed PPE cases had a CD4 cell count less than 350 cells/µL.

During the second phase of the study, 149 patients were screened and enrolled as controls during a one-week period in October 2008. A comparison of demographic characteristics amongst the confirmed PPE cases (*n*=42) versus the controls (*n*=149) resulted in no difference between the two groups, including age and sex (Table 3).

**Table 3:** Demographic characteristics of subjects enrolled in the study^a^

Characteristic	PPE cases (*n*=42)	Controls (*n*=149)	*p*
Age, mean years (SD)	34.2 (7.5)	33.4 (7.7)	0.57
Female, number (%)	29 (69.0)	86 (57.7)	0.185
Rural residence, number (%)	25 (59.5)	86 (57.7)	0.834
Time since HIV diagnosis, mean days (SD)	834.4 (735.5)	699.5 (685.7)	0.28
Median CD4, most recent (interquartile range)	225.5 (105.5–490.3)	425 (212.0–641.0)	<0.000
Patients receiving CTZ, number (%)	38 (90.4)	148 (99.3)	0.002

a Characteristics of study participants: PPE are cases defined as adults experiencing a pruritic skin eruption for longer than one month duration, with evidence of multiple papular or nodular lesions and a skin biopsy indicating an “insect bite reaction.” Controls are defined as adults with no active skin rash.

The difference in the number of females amongst the two groups was not significant (cases 69% vs. controls 57.7%; *p*=0.185), and there was no statistical difference within the proportion of cases versus controls who live in a rural setting (*n*=25, 59.5% vs. *n*=86, 57.7%; *p*=0.834). In addition, there was no significant difference in the duration since HIV diagnosis between the two groups (2.3 years, SD 735.5 vs. 1.9 years, SD 1.9; *p*=0.28). Subjects with PPE, however, had significantly lower CD4 cell counts in comparison to the controls (median, 225.5 cells/µL [interquartile range, or IQR: 105.5–490.3 cells/µL] vs. 425 cells/µL [IQR, 212–641 cells/µL]; *p*=0.0001).

We compared environmental exposures amongst the PPE cases versus the controls, and found similar findings for history of mosquito bites, use of mosquito netting, exposure to animals, and work outdoors (Table 4).

**Table 4:** Comparison of environmental exposures amongst PPE cases and controls

Exposure	Cases (*n*=42)	Controls (*n*=149)	Odds ratio, 95% CI
Female, number (%)	29 (69.05)	86 (57.72)	1.63, 0.79–3.39
History of mosquito bites, number (%)	34 (81.0)	131 (87.9)	0.584, 0.23–1.46
Use of mosquito netting, number (%)	5 (11.9)	27 (18.1)	0.61, 0.2–1.70
Exposure to animals, number (%)	11 (26.2)	47 (31.5)	0.77, 0.36–1.70
Work outdoors, number (%)	20 (50.0)	62 (41.9)	0.72, 0.36–1.50
Non-usage of insect repellent, coils, or spray, number (%)	29 (69.0)	64 (43.0)	2.96, 1.43–6.15

Patients with PPE, however, were less likely to use insect repellents, coils, and/or sprays in comparison to controls.

## Discussion

In the 1466 routinely evaluated HIV-positive patients at GHTM, we histologically confirmed PPE in 42 individuals (2.9%). EF, in comparison, is slightly higher in prevalence [19,24]. Of patients who presented with bilateral, symmetric, pruritic papules on the extremities for more than one month duration (*n*=52), 42 (79.2%) had histologic confirmation of PPE. This revalidation of the criteria used in the 2004 Ugandan PPE study indicates that we may use this clinical scenario as a screening technique in the diagnosis of PPE.

Using the rash severity scale, the severity of skin rash was not significantly associated with the CD4 count. This is a somewhat different result from those of previous studies and implies that we cannot use rash distribution or severity as an indicator of CD4 count.

CD4 counts in our PPE study population are higher on average than what has been previously reported [5–7]. This may be related to patients presenting to GHTM for HIV care at earlier stages of the disease. GHTM has been providing HIV testing, care, outreach, and treatment to patients in India since 2004. Given this history, patients with HIV may present earlier after initial infection with HIV and prior to the development of severe HIV/AIDS. While CD4 counts in our case population were higher than previously reported [5–7], the majority were still less than CD4 counts of 350 cells/µL. European and US guidelines have recommended that ARV therapy be initiated in HIV-positive patients with CD4 counts less than 350 cells/µL [24].

No studies to date have examined potential PPE-associated risk factors in HIV-positive persons affected with PPE in comparison to a control group. This study attempted to describe the profile of patients with and without PPE who were presenting to GHTM from the same areas of India with similar environmental exposures. Overall, a small percentage of HIV-positive patients presented with PPE. However, because the skin allows for easy visual diagnoses, PPE may be considered a surrogate marker of immunosuppression and an indicator for the need to initiate ARV therapy in resource-poor settings where CD4 count cannot be assessed.

In addition, protective measures against mosquito bites such as repellents, sprays, and coils appeared to be important in preventing PPE in subjects at risk. Increased advocacy, availability, and use may lower the disease burden. Mosquito netting did not seem to play a role, although it is not commonly used in India so its actual impact is difficult to accurately determine through this study. This may be explained by the fact that subjects are being bitten throughout the day as well as the evening, and that nets are protective only against malaria.

Patients living in rural versus urban settings were not found to have any statistically significant difference in their risk for PPE, but more in-depth surveys of the environment to include nearby water pools, animals, and topography may help identify patients who are at risk of exposure to arthropods. Future studies from other areas where PPE is prevalent will be helpful in further defining environmental risk factors. Treatment options, response to ARV therapy, outcome in PPE patients, and recurrent PPE during ARV treatment are areas that require additional study. Genetic polymorphisms may also be an area of future study to explain why certain HIV-positive patients develop PPE while others do not.

Of note, cases and controls were collected at different times, and therefore the effect of seasonal variation needs to be considered in this study. Controls were collected during the early phases of the rainy season (i.e. November) when there are potentially more insects, representing a bias toward controls having more PPE versus cases. The reverse was true in our study. However, it could be argued that subjects may have taken greater protective measures against being affected by arthropod bites during the rainy season (e.g. slept under mosquito netting, worn protective spray, or use lighted antimosquito coils), resulting in a lower exposure to such arthropods.

## Conclusions

In conclusion, the majority of biopsies in persons who presented with bilateral, symmetric, pruritic papules on the extremities with duration longer than one month showed histology consistent with confirmed PPE. In addition, this finding was associated with CD4 counts lower than 350 cells/µL, at which point ARV treatment initiation is recommended as per international standards [25]. PPE may therefore be used as a marker for initiating therapy in resource-poor settings. Environmental factors are still to be elucidated, but protective measures against mosquito bites such as the use of mosquito coils appeared to be beneficial against developing PPE.
